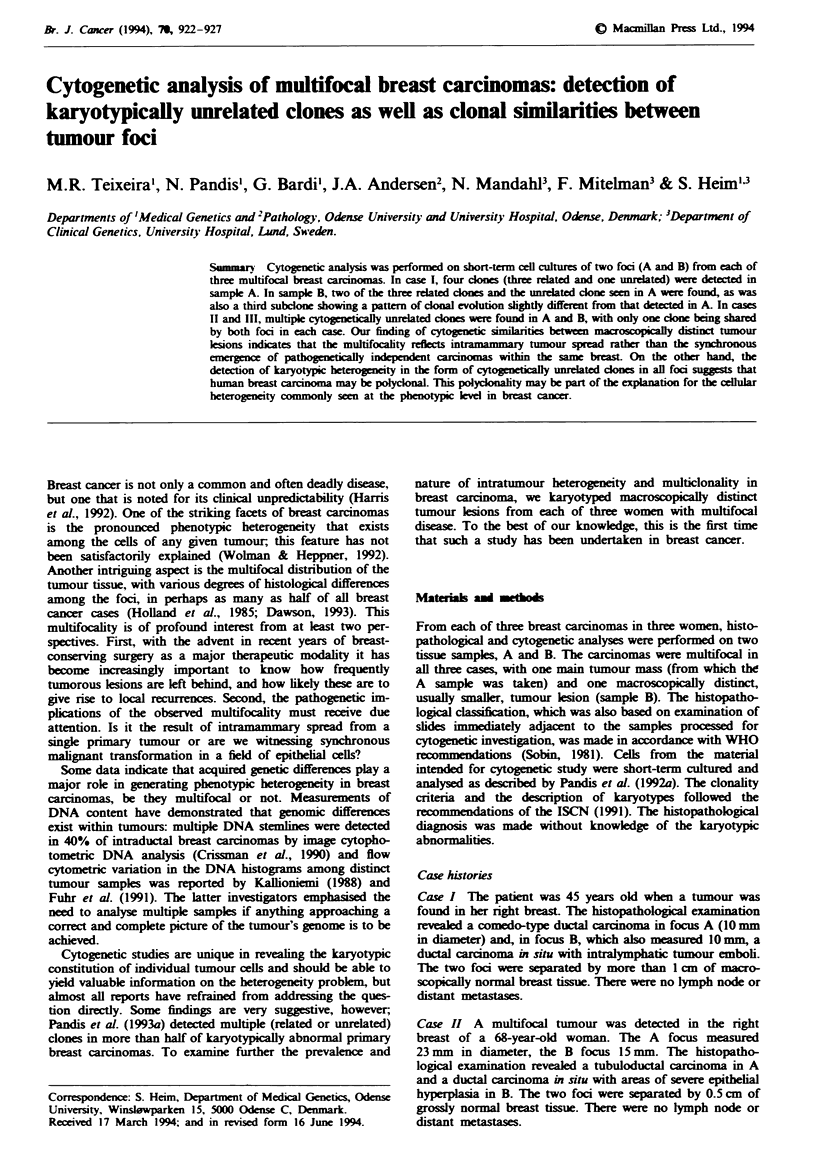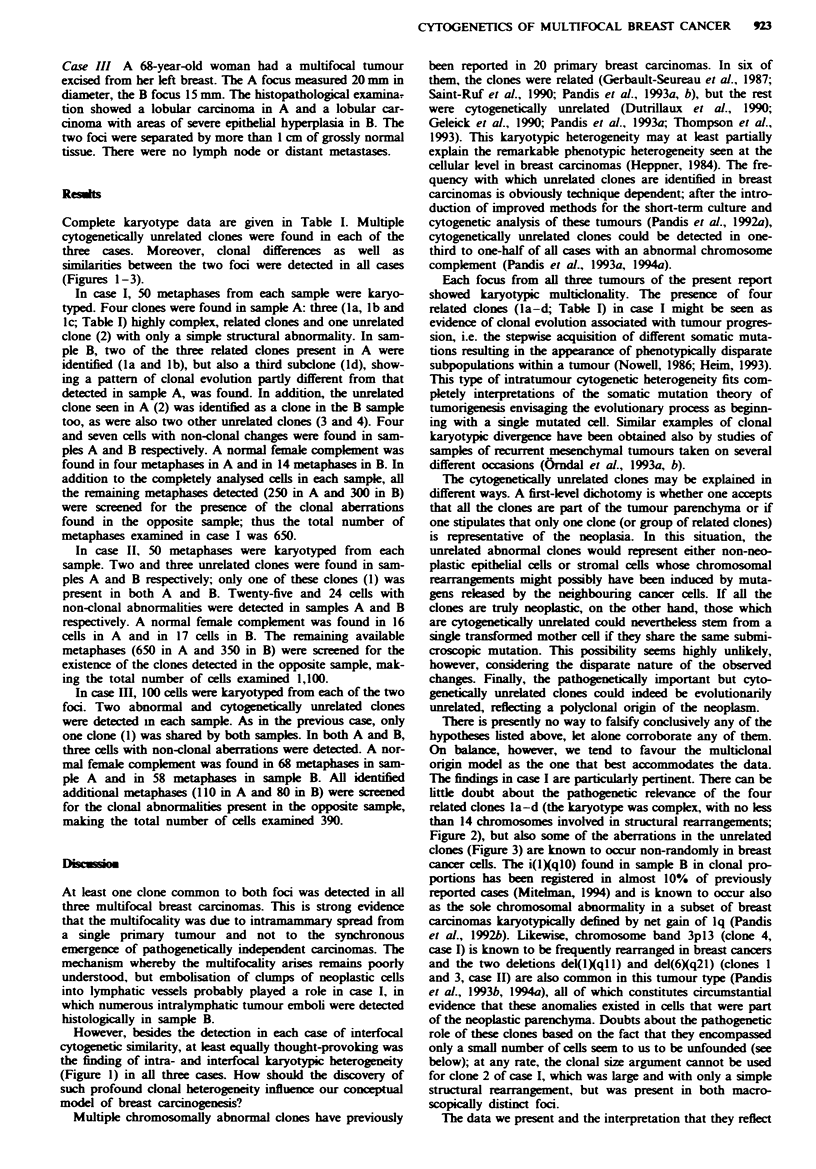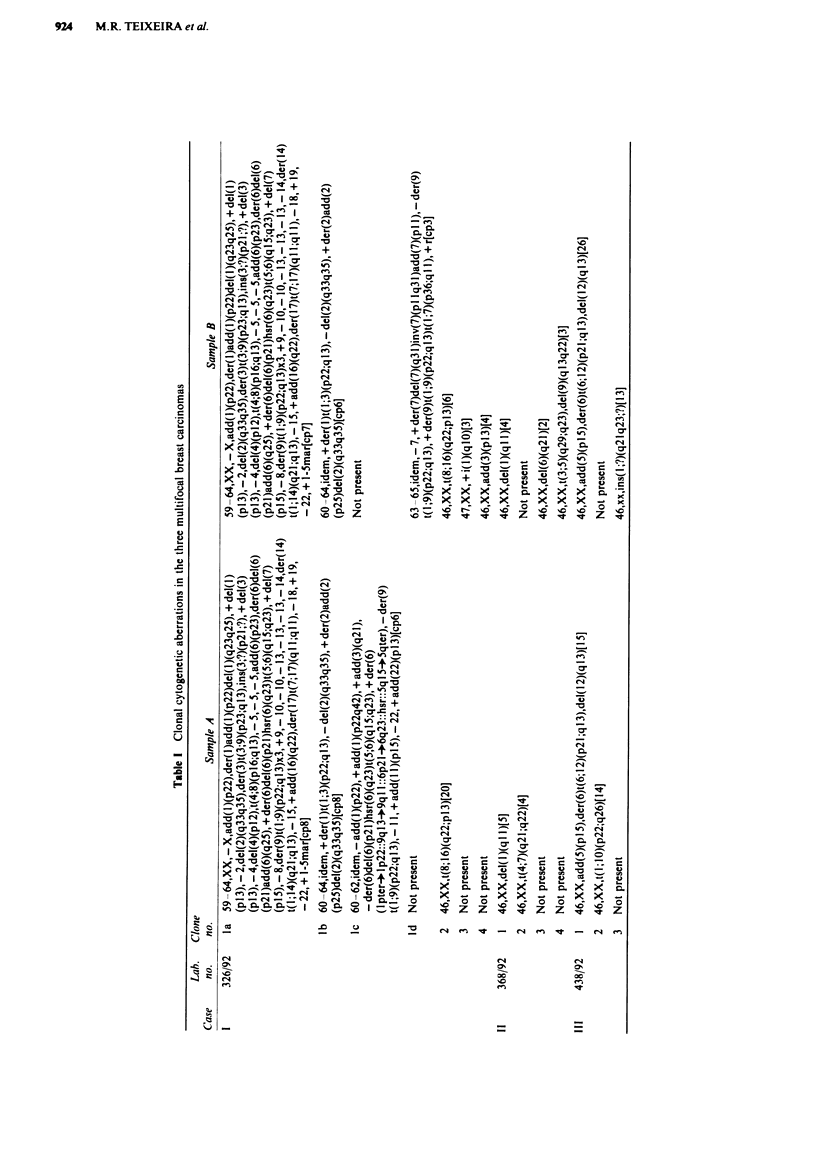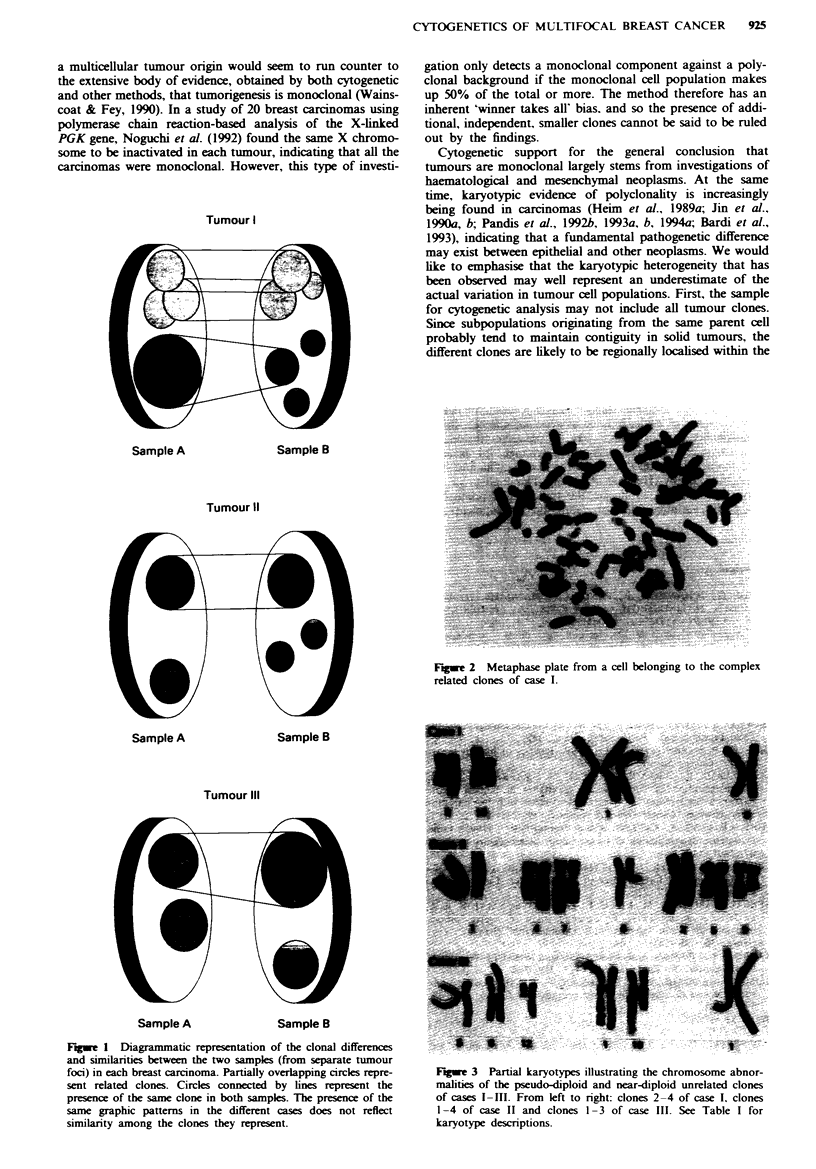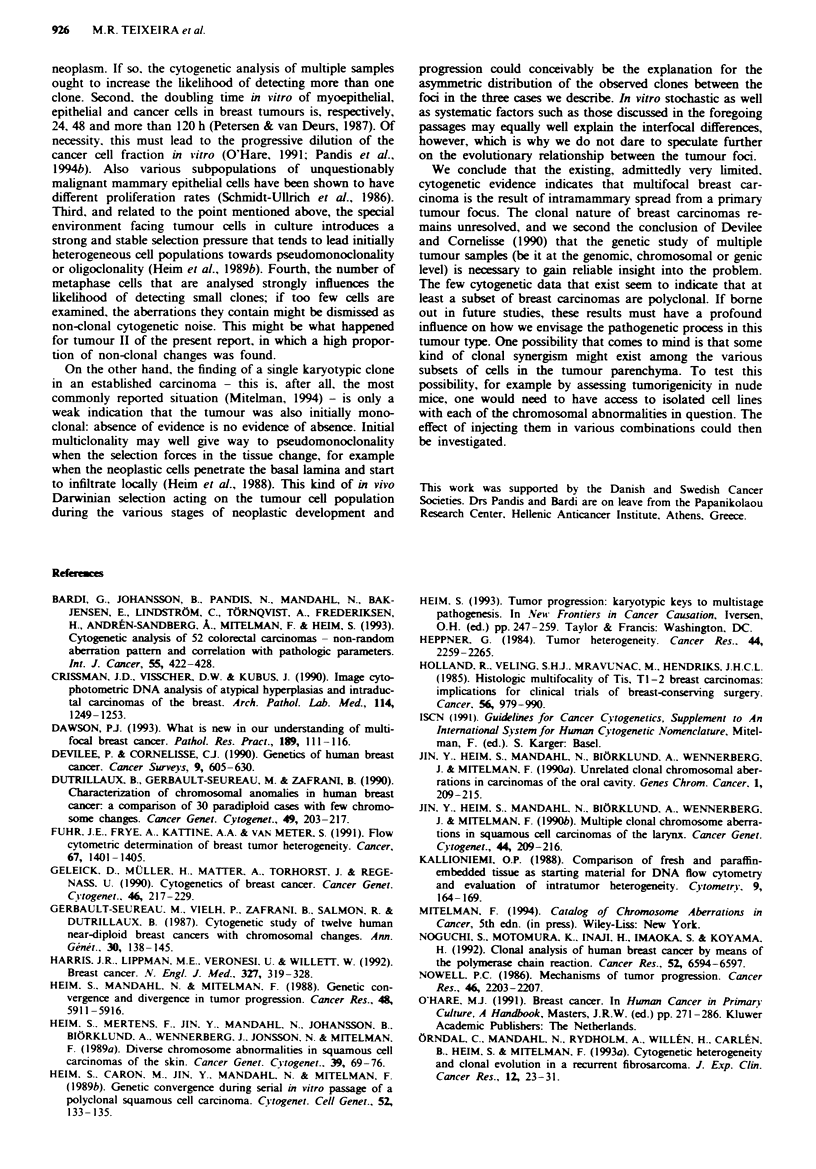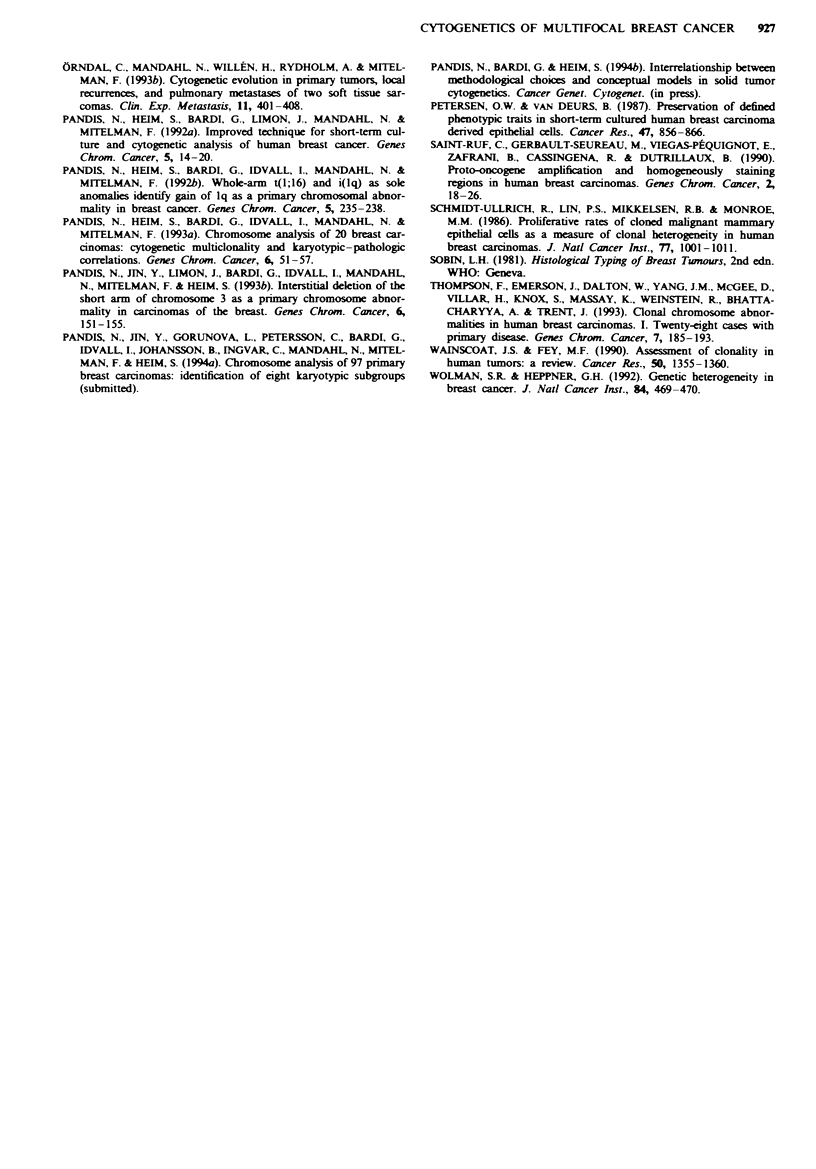# Cytogenetic analysis of multifocal breast carcinomas: detection of karyotypically unrelated clones as well as clonal similarities between tumour foci.

**DOI:** 10.1038/bjc.1994.421

**Published:** 1994-11

**Authors:** M. R. Teixeira, N. Pandis, G. Bardi, J. A. Andersen, N. Mandahl, F. Mitelman, S. Heim

**Affiliations:** Department of Medical Genetics, Odense University, Denmark.

## Abstract

**Images:**


Br. J. Cancer (1994), 70, 922-927

© Macmillan Press Ltd., 1994

### Cytogenetic analysis of multifocal breast carcinomas: detection of karyotypically unrelated clones as well as clonal similarities between tumour foci

M.R. Teixeira[1], N. Pandis[1], G. Bardi[1], J.A. Andersen[2], N. Mandahl[3], F. Mitelman[3] & S. Heim[1,3]

*Departments of [1]Medical Genetics and [2]Pathology, Odense University and University Hospital, Odense, Denmark; [3]Department of Clinical Genetics, University Hospital, Lund, Sweden.*

**Summary** Cytogenetic analysis was performed on short-term cell cultures of two foci (A and B) from each of three multifocal breast carcinomas. In case I, four clones (three related and one unrelated) were detected in sample A. In sample B, two of the three related clones and the unrelated clone seen in A were found, as was also a third subclone showing a pattern of clonal evolution slightly different from that detected in A. In cases II and III, multiple cytogenetically unrelated clones were found in A and B, with only one clone being shared by both foci in each case. Our finding of cytogenetic similarities between macroscopically distinct tumour lesions indicates that the multifocality reflects intramammary tumour spread rather than the synchronous emergence of pathogenetically independent carcinomas within the same breast. On the other hand, the detection of karyotypic heterogeneity in the form of cytogenetically unrelated clones in all foci suggests that human breast carcinoma may be polyclonal. This polyclonality may be part of the explanation for the cellular heterogeneity commonly seen at the phenotypic level in breast cancer.

Breast cancer is not only a common and often deadly disease, but one that is noted for its clinical unpredictability (Harris *et al.*, 1992). One of the striking facets of breast carcinomas is the pronounced phenotypic heterogeneity that exists among the cells of any given tumour; this feature has not been satisfactorily explained (Wolman & Heppner, 1992). Another intriguing aspect is the multifocal distribution of the tumour tissue, with various degrees of histological differences among the foci, in perhaps as many as half of all breast cancer cases (Holland *et al.*, 1985; Dawson, 1993). This multifocality is of profound interest from at least two perspectives. First, with the advent in recent years of breast-conserving surgery as a major therapeutic modality it has become increasingly important to know how frequently tumorous lesions are left behind, and how likely these are to give rise to local recurrences. Second, the pathogenetic implications of the observed multifocality must receive due attention. Is it the result of intramammary spread from a single primary tumour or are we witnessing synchronous malignant transformation in a field of epithelial cells?

Some data indicate that acquired genetic differences play a major role in generating phenotypic heterogeneity in breast carcinomas, be they multifocal or not. Measurements of DNA content have demonstrated that genomic differences exist within tumours: multiple DNA stemlines were detected in 40% of intraductal breast carcinomas by image cytophotometric DNA analysis (Crissman *et al.*, 1990) and flow cytometric variation in the DNA histograms among distinct tumour samples was reported by Kallioniemi (1988) and Fuhr *et al.* (1991). The latter investigators emphasised the need to analyse multiple samples if anything approaching a correct and complete picture of the tumour's genome is to be achieved.

Cytogenetic studies are unique in revealing the karyotypic constitution of individual tumour cells and should be able to yield valuable information on the heterogeneity problem, but almost all reports have refrained from addressing the question directly. Some findings are very suggestive, however; Pandis *et al.* (1993*a*) detected multiple (related or unrelated) clones in more than half of karyotypically abnormal primary breast carcinomas. To examine further the prevalence and nature of intratumour heterogeneity and multiclonality in breast carcinoma, we karyotyped macroscopically distinct tumour lesions from each of three women with multifocal disease. To the best of our knowledge, this is the first time that such a study has been undertaken in breast cancer.

### Materials and methods

From each of three breast carcinomas in three women, histopathological and cytogenetic analyses were performed on two tissue samples, A and B. The carcinomas were multifocal in all three cases, with one main tumour mass (from which the A sample was taken) and one macroscopically distinct, usually smaller, tumour lesion (sample B). The histopathological classification, which was also based on examination of slides immediately adjacent to the samples processed for cytogenetic investigation, was made in accordance with WHO recommendations (Sobin, 1981). Cells from the material intended for cytogenetic study were short-term cultured and analysed as described by Pandis *et al.* (1992*a*). The clonality criteria and the description of karyotypes followed the recommendations of the ISCN (1991). The histopathological diagnosis was made without knowledge of the karyotypic abnormalities.

### *Case histories*

*Case I* The patient was 45 years old when a tumour was found in her right breast. The histopathological examination revealed a comedo-type ductal carcinoma in focus A (10 mm in diameter) and, in focus B, which also measured 10 mm, a ductal carcinoma *in situ* with intralymphatic tumour emboli. The two foci were separated by more than 1 cm of macroscopically normal breast tissue. There were no lymph node or distant metastases.

*Case II* A multifocal tumour was detected in the right breast of a 68-year-old woman. The A focus measured 23 mm in diameter, the B focus 15 mm. The histopathological examination revealed a tubuloductal carcinoma in A and a ductal carcinoma *in situ* with areas of severe epithelial hyperplasia in B. The two foci were separated by 0.5 cm of grossly normal breast tissue. There were no lymph node or distant metastases.

Correspondence: S. Heim, Department of Medical Genetics, Odense University, Winsløwparken 15, 5000 Odense C, Denmark.
Received 17 March 1994; and in revised form 16 June 1994.

*Case III* A 68-year-old woman had a multifocal tumour excised from her left breast. The A focus measured 20 mm in diameter, the B focus 15 mm. The histopathological examination showed a lobular carcinoma in A and a lobular carcinoma with areas of severe epithelial hyperplasia in B. The two foci were separated by more than 1 cm of grossly normal tissue. There were no lymph node or distant metastases.

### Results

Complete karyotype data are given in Table I. Multiple cytogenetically unrelated clones were found in each of the three cases. Moreover, clonal differences as well as similarities between the two foci were detected in all cases (Figures 1–3).

In case I, 50 metaphases from each sample were karyotyped. Four clones were found in sample A: three (1a, 1b and 1c; Table I) highly complex, related clones and one unrelated clone (2) with only a simple structural abnormality. In sample B, two of the three related clones present in A were identified (1a and 1b), but also a third subclone (1d), showing a pattern of clonal evolution partly different from that detected in sample A, was found. In addition, the unrelated clone seen in A (2) was identified as a clone in the B sample too, as were also two other unrelated clones (3 and 4). Four and seven cells with non-clonal changes were found in samples A and B respectively. A normal female complement was found in four metaphases in A and in 14 metaphases in B. In addition to the completely analysed cells in each sample, all the remaining metaphases detected (250 in A and 300 in B) were screened for the presence of the clonal aberrations found in the opposite sample; thus the total number of metaphases examined in case I was 650.

In case II, 50 metaphases were karyotyped from each sample. Two and three unrelated clones were found in samples A and B respectively; only one of these clones (1) was present in both A and B. Twenty-five and 24 cells with non-clonal abnormalities were detected in samples A and B respectively. A normal female complement was found in 16 cells in A and in 17 cells in B. The remaining available metaphases (650 in A and 350 in B) were screened for the existence of the clones detected in the opposite sample, making the total number of cells examined 1,100.

In case III, 100 cells were karyotyped from each of the two foci. Two abnormal and cytogenetically unrelated clones were detected in each sample. As in the previous case, only one clone (1) was shared by both samples. In both A and B, three cells with non-clonal aberrations were detected. A normal female complement was found in 68 metaphases in sample A and in 58 metaphases in sample B. All identified additional metaphases (110 in A and 80 in B) were screened for the clonal abnormalities present in the opposite sample, making the total number of cells examined 390.

### Discussion

At least one clone common to both foci was detected in all three multifocal breast carcinomas. This is strong evidence that the multifocality was due to intramammary spread from a single primary tumour and not to the synchronous emergence of pathogenetically independent carcinomas. The mechanism whereby the multifocality arises remains poorly understood, but embolisation of clumps of neoplastic cells into lymphatic vessels probably played a role in case I, in which numerous intralymphatic tumour emboli were detected histologically in sample B.

However, besides the detection in each case of interfocal cytogenetic similarity, at least equally thought-provoking was the finding of intra- and interfocal karyotypic heterogeneity (Figure 1) in all three cases. How should the discovery of such profound clonal heterogeneity influence our conceptual model of breast carcinogenesis?

Multiple chromosomally abnormal clones have previously been reported in 20 primary breast carcinomas. In six of them, the clones were related (Gerbault-Seureau *et al*., 1987; Saint-Ruf *et al*., 1990; Pandis *et al*., 1993*a*, *b*), but the rest were cytogenetically unrelated (Dutrillaux *et al*., 1990; Geleick *et al*., 1990; Pandis *et al*., 1993*a*; Thompson *et al*., 1993). This karyotypic heterogeneity may at least partially explain the remarkable phenotypic heterogeneity seen at the cellular level in breast carcinomas (Heppner, 1984). The frequency with which unrelated clones are identified in breast carcinomas is obviously technique dependent; after the introduction of improved methods for the short-term culture and cytogenetic analysis of these tumours (Pandis *et al*., 1992*a*), cytogenetically unrelated clones could be detected in one-third to one-half of all cases with an abnormal chromosome complement (Pandis *et al*., 1993*a*, 1994*a*).

Each focus from all three tumours of the present report showed karyotypic multiclonality. The presence of four related clones (1a–d; Table I) in case I might be seen as evidence of clonal evolution associated with tumour progression, i.e. the stepwise acquisition of different somatic mutations resulting in the appearance of phenotypically disparate subpopulations within a tumour (Nowell, 1986; Heim, 1993). This type of intratumour cytogenetic heterogeneity fits completely interpretations of the somatic mutation theory of tumorigenesis envisaging the evolutionary process as beginning with a single mutated cell. Similar examples of clonal karyotypic divergence have been obtained also by studies of samples of recurrent mesenchymal tumours taken on several different occasions (Örndal *et al*., 1993*a*, *b*).

The cytogenetically unrelated clones may be explained in different ways. A first-level dichotomy is whether one accepts that all the clones are part of the tumour parenchyma or if one stipulates that only one clone (or group of related clones) is representative of the neoplasia. In this situation, the unrelated abnormal clones would represent either non-neoplastic epithelial cells or stromal cells whose chromosomal rearrangements might possibly have been induced by mutagens released by the neighbouring cancer cells. If all the clones are truly neoplastic, on the other hand, those which are cytogenetically unrelated could nevertheless stem from a single transformed mother cell if they share the same submicroscopic mutation. This possibility seems highly unlikely, however, considering the disparate nature of the observed changes. Finally, the pathogenetically important but cytogenetically unrelated clones could indeed be evolutionarily unrelated, reflecting a polyclonal origin of the neoplasm.

There is presently no way to falsify conclusively any of the hypotheses listed above, let alone corroborate any of them. On balance, however, we tend to favour the multiclonal origin model as the one that best accommodates the data. The findings in case I are particularly pertinent. There can be little doubt about the pathogenetic relevance of the four related clones 1a–d (the karyotype was complex, with no less than 14 chromosomes involved in structural rearrangements; Figure 2), but also some of the aberrations in the unrelated clones (Figure 3) are known to occur non-randomly in breast cancer cells. The i(1)(q10) found in sample B in clonal proportions has been registered in almost 10% of previously reported cases (Mitelman, 1994) and is known to occur also as the sole chromosomal abnormality in a subset of breast carcinomas karyotypically defined by net gain of 1q (Pandis *et al*., 1992*b*). Likewise, chromosome band 3p13 (clone 4, case I) is known to be frequently rearranged in breast cancers and the two deletions del(1)(q11) and del(6)(q21) (clones 1 and 3, case II) are also common in this tumour type (Pandis *et al*., 1993*b*, 1994*a*), all of which constitutes circumstantial evidence that these anomalies existed in cells that were part of the neoplastic parenchyma. Doubts about the pathogenetic role of these clones based on the fact that they encompassed only a small number of cells seem to us to be unfounded (see below); at any rate, the clonal size argument cannot be used for clone 2 of case I, which was large and with only a simple structural rearrangement, but was present in both macroscopically distinct foci.

The data we present and the interpretation that they reflect

**Table I** Clonal cytogenetic aberrations in the three multifocal breast carcinomas

| Case | Lab. no. | Clone no. | Sample A | Sample B |
|---|---|---|---|---|
| I | 326/92 | 1a | 59–64,XX,−X,add(1)(p22),der(1)add(1)(p22)del(1)(q23q25),+del(1)(p13),−2,del(2)(q33q35),der(3)t(3;9)(p23;q13),ins(3;?)(p21;?),+del(3)(p13),−4,del(4)(p12),t(4;8)(p16;q13),−5,−5,−5,add(6)(p23),der(6)del(6)(p21)add(6)(q25),+der(6)del(6)(p21)hsr(6)(q23)t(5;6)(q15;q23),+del(7)(p15),−8,der(9)t(1;9)(p22;q13)x3,+9,−10,−10,−13,−13,−13,−14,der(14)t(1;14)(q21;q13),−15,+add(16)(q22),der(17)t(7;17)(q11;q11),−18,+19,−22,+1-5mar[cp8] | 59–64,XX,−X,add(1)(p22),der(1)add(1)(p22)del(1)(q23q25),+del(1)(p13),−2,del(2)(q33q35),der(3)t(3;9)(p23;q13),ins(3;?)(p21;?),+del(3)(p13),−4,del(4)(p12),t(4;8)(p16;q13),−5,−5,−5,add(6)(p23),der(6)del(6)(p21)add(6)(q25),+der(6)del(6)(p21)hsr(6)(q23)t(5;6)(q15;q23),+del(7)(p15),−8,der(9)t(1;9)(p22;q13)x3,+9,−10,−10,−13,−13,−13,−14,der(14)t(1;14)(q21;q13),−15,+add(16)(q22),der(17)t(7;17)(q11;q11),−18,+19,−22,+1-5mar[cp7] |
| | | 1b | 60–64,idem,+der(1)t(1;3)(p22;q13),−del(2)(q33q35),+der(2)add(2)(p25)del(2)(q33q35)[cp8] | 60–64,idem,+der(1)t(1;3)(p22;q13),−del(2)(q33q35),+der(2)add(2)(p25)del(2)(q33q35)[cp6] |
| | | 1c | 60–62,idem,−add(1)(p22),+add(1)(p22q42),+add(3)(q21),−der(6)del(6)(p21)hsr(6)(q23)t(5;6)(q15;q23),+der(6)(1pter→1p22::9q13→9q11::6p21→6q23::hsr::5q15→5qter),−der(9)t(1;9)(p22;q13),−11,+add(11)(p15),−22,+add(22)(p13)[cp6] | Not present |
| | | 1d | Not present | 63–65,idem,−7,+der(7)del(7)(q31)inv(7)(p11q31)add(7)(p11),−der(9)t(1;9)(p22;q13),+der(9)t(1;9)(p22;q13)t(1;7)(p36;q11),+r[cp3] |
| | | 2 | 46,XX,t(8;16)(q22;p13)[20] | 46,XX,t(8;16)(q22;p13)[6] |
| | | 3 | Not present | 47,XX,+i(1)(q10)[3] |
| | | 4 | Not present | 46,XX,add(3)(p13)[4] |
| II | 368/92 | 1 | 46,XX,del(1)(q11)[5] | 46,XX,del(1)(q11)[4] |
| | | 2 | 46,XX,t(4;7)(q21;q22)[4] | Not present |
| | | 3 | Not present | 46,XX,del(6)(q21)[2] |
| | | 4 | Not present | 46,XX,t(3;5)(q29;q23),del(9)(q13q22)[3] |
| III | 438/92 | 1 | 46,XX,add(5)(p15),der(6)t(6;12)(p21;q13),del(12)(q13)[15] | 46,XX,add(5)(p15),der(6)t(6;12)(p21;q13),del(12)(q13)[26] |
| | | 2 | 46,XX,t(1;10)(p22;q26)[14] | Not present |
| | | 3 | Not present | 46,xx,ins(1;?)(q21q23;?)[13] |

a multicellular tumour origin would seem to run counter to the extensive body of evidence, obtained by both cytogenetic and other methods, that tumorigenesis is monoclonal (Wainscoat & Fey, 1990). In a study of 20 breast carcinomas using polymerase chain reaction-based analysis of the X-linked *PGK* gene, Noguchi *et al.* (1992) found the same X chromosome to be inactivated in each tumour, indicating that all the carcinomas were monoclonal. However, this type of investigation only detects a monoclonal component against a polyclonal background if the monoclonal cell population makes up 50% of the total or more. The method therefore has an inherent 'winner takes all' bias, and so the presence of additional, independent, smaller clones cannot be said to be ruled out by the findings.

Cytogenetic support for the general conclusion that tumours are monoclonal largely stems from investigations of haematological and mesenchymal neoplasms. At the same time, karyotypic evidence of polyclonality is increasingly being found in carcinomas (Heim *et al.*, 1989*a*; Jin *et al.*, 1990*a*, *b*; Pandis *et al.*, 1992*b*, 1993*a*, *b*, 1994*a*; Bardi *et al.*, 1993), indicating that a fundamental pathogenetic difference may exist between epithelial and other neoplasms. We would like to emphasise that the karyotypic heterogeneity that has been observed may well represent an underestimate of the actual variation in tumour cell populations. First, the sample for cytogenetic analysis may not include all tumour clones. Since subpopulations originating from the same parent cell probably tend to maintain contiguity in solid tumours, the different clones are likely to be regionally localised within the

**Figure 1** Diagrammatic representation of the clonal differences and similarities between the two samples (from separate tumour foci) in each breast carcinoma. Partially overlapping circles represent related clones. Circles connected by lines represent the presence of the same clone in both samples. The presence of the same graphic patterns in the different cases does not reflect similarity among the clones they represent.

**Figure 2** Metaphase plate from a cell belonging to the complex related clones of case I.

**Figure 3** Partial karyotypes illustrating the chromosome abnormalities of the pseudo-diploid and near-diploid unrelated clones of cases I–III. From left to right: clones 2–4 of case I, clones 1–4 of case II and clones 1–3 of case III. See Table I for karyotype descriptions.

neoplasm. If so, the cytogenetic analysis of multiple samples ought to increase the likelihood of detecting more than one clone. Second, the doubling time *in vitro* of myoepithelial, epithelial and cancer cells in breast tumours is, respectively, 24, 48 and more than 120 h (Petersen & van Deurs, 1987). Of necessity, this must lead to the progressive dilution of the cancer cell fraction *in vitro* (O'Hare, 1991; Pandis *et al.*, 1994*b*). Also various subpopulations of unquestionably malignant mammary epithelial cells have been shown to have different proliferation rates (Schmidt-Ullrich *et al.*, 1986). Third, and related to the point mentioned above, the special environment facing tumour cells in culture introduces a strong and stable selection pressure that tends to lead initially heterogeneous cell populations towards pseudomonoclonality or oligoclonality (Heim *et al.*, 1989*b*). Fourth, the number of metaphase cells that are analysed strongly influences the likelihood of detecting small clones; if too few cells are examined, the aberrations they contain might be dismissed as non-clonal cytogenetic noise. This might be what happened for tumour II of the present report, in which a high proportion of non-clonal changes was found.

On the other hand, the finding of a single karyotypic clone in an established carcinoma – this is, after all, the most commonly reported situation (Mitelman, 1994) – is only a weak indication that the tumour was also initially monoclonal: absence of evidence is no evidence of absence. Initial multiclonality may well give way to pseudomonoclonality when the selection forces in the tissue change, for example when the neoplastic cells penetrate the basal lamina and start to infiltrate locally (Heim *et al.*, 1988). This kind of *in vivo* Darwinian selection acting on the tumour cell population during the various stages of neoplastic development and progression could conceivably be the explanation for the asymmetric distribution of the observed clones between the foci in the three cases we describe. *In vitro* stochastic as well as systematic factors such as those discussed in the foregoing passages may equally well explain the interfocal differences, however, which is why we do not dare to speculate further on the evolutionary relationship between the tumour foci.

We conclude that the existing, admittedly very limited, cytogenetic evidence indicates that multifocal breast carcinoma is the result of intramammary spread from a primary tumour focus. The clonal nature of breast carcinomas remains unresolved, and we second the conclusion of Devilee and Cornelisse (1990) that the genetic study of multiple tumour samples (be it at the genomic, chromosomal or genic level) is necessary to gain reliable insight into the problem. The few cytogenetic data that exist seem to indicate that at least a subset of breast carcinomas are polyclonal. If borne out in future studies, these results must have a profound influence on how we envisage the pathogenetic process in this tumour type. One possibility that comes to mind is that some kind of clonal synergism might exist among the various subsets of cells in the tumour parenchyma. To test this possibility, for example by assessing tumorigenicity in nude mice, one would need to have access to isolated cell lines with each of the chromosomal abnormalities in question. The effect of injecting them in various combinations could then be investigated.

This work was supported by the Danish and Swedish Cancer Societies. Drs Pandis and Bardi are on leave from the Papanikolaou Research Center, Hellenic Anticancer Institute, Athens, Greece.

### References

BARDI, G., JOHANSSON, B., PANDIS, N., MANDAHL, N., BAK-JENSEN, E., LINDSTRÖM, C., TÖRNQVIST, A., FREDERIKSEN, H., ANDRÉN-SANDBERG, Å., MITELMAN, F. & HEIM, S. (1993). Cytogenetic analysis of 52 colorectal carcinomas – non-random aberration pattern and correlation with pathologic parameters. *Int. J. Cancer*, **55**, 422–428.

CRISSMAN, J.D., VISSCHER, D.W. & KUBUS, J. (1990). Image cytophotometric DNA analysis of atypical hyperplasias and intraductal carcinomas of the breast. *Arch. Pathol. Lab. Med.*, **114**, 1249–1253.

DAWSON, P.J. (1993). What is new in our understanding of multifocal breast cancer. *Pathol. Res. Pract.*, **189**, 111–116.

DEVILEE, P. & CORNELISSE, C.J. (1990). Genetics of human breast cancer. *Cancer Surveys*, **9**, 605–630.

DUTRILLAUX, B., GERBAULT-SEUREAU, M. & ZAFRANI, B. (1990). Characterization of chromosomal anomalies in human breast cancer: a comparison of 30 paradiploid cases with few chromosome changes. *Cancer Genet. Cytogenet.*, **49**, 203–217.

FUHR, J.E., FRYE, A., KATTINE, A.A. & VAN METER, S. (1991). Flow cytometric determination of breast tumor heterogeneity. *Cancer*, **67**, 1401–1405.

GELEICK, D., MÜLLER, H., MATTER, A., TORHORST, J. & REGENASS, U. (1990). Cytogenetics of breast cancer. *Cancer Genet. Cytogenet.*, **46**, 217–229.

GERBAULT-SEUREAU, M., VIELH, P., ZAFRANI, B., SALMON, R. & DUTRILLAUX, B. (1987). Cytogenetic study of twelve human near-diploid breast cancers with chromosomal changes. *Ann. Génét.*, **30**, 138–145.

HARRIS, J.R., LIPPMAN, M.E., VERONESI, U. & WILLETT, W. (1992). Breast cancer. *N. Engl. J. Med.*, **327**, 319–328.

HEIM, S., MANDAHL, N. & MITELMAN, F. (1988). Genetic convergence and divergence in tumor progression. *Cancer Res.*, **48**, 5911–5916.

HEIM, S., MERTENS, F., JIN, Y., MANDAHL, N., JOHANSSON, B., BIÖRKLUND, A., WENNERBERG, J., JONSSON, N. & MITELMAN, F. (1989*a*). Diverse chromosome abnormalities in squamous cell carcinomas of the skin. *Cancer Genet. Cytogenet.*, **39**, 69–76.

HEIM, S., CARON, M., JIN, Y., MANDAHL, N. & MITELMAN, F. (1989*b*). Genetic convergence during serial *in vitro* passage of a polyclonal squamous cell carcinoma. *Cytogenet. Cell Genet.*, **52**, 133–135.

HEIM, S. (1993). Tumor progression: karyotypic keys to multistage pathogenesis. In *New Frontiers in Cancer Causation*, Iversen, O.H. (ed.) pp. 247–259. Taylor & Francis: Washington, DC.

HEPPNER, G. (1984). Tumor heterogeneity. *Cancer Res.*, **44**, 2259–2265.

HOLLAND, R., VELING, S.H.J., MRAVUNAC, M., HENDRIKS, J.H.C.L. (1985). Histologic multifocality of Tis, T1–2 breast carcinomas: implications for clinical trials of breast-conserving surgery. *Cancer*, **56**, 979–990.

ISCN (1991). *Guidelines for Cancer Cytogenetics, Supplement to An International System for Human Cytogenetic Nomenclature*, Mitelman, F. (ed.). S. Karger: Basel.

JIN, Y., HEIM, S., MANDAHL, N., BIÖRKLUND, A., WENNERBERG, J. & MITELMAN, F. (1990*a*). Unrelated clonal chromosomal aberrations in carcinomas of the oral cavity. *Genes Chrom. Cancer*, **1**, 209–215.

JIN, Y., HEIM, S., MANDAHL, N., BIÖRKLUND, A., WENNERBERG, J. & MITELMAN, F. (1990*b*). Multiple clonal chromosome aberrations in squamous cell carcinomas of the larynx. *Cancer Genet. Cytogenet.*, **44**, 209–216.

KALLIONIEMI, O.P. (1988). Comparison of fresh and paraffin-embedded tissue as starting material for DNA flow cytometry and evaluation of intratumor heterogeneity. *Cytometry*, **9**, 164–169.

MITELMAN, F. (1994). *Catalog of Chromosome Aberrations in Cancer*, 5th edn. (in press). Wiley-Liss: New York.

NOGUCHI, S., MOTOMURA, K., INAJI, H., IMAOKA, S. & KOYAMA, H. (1992). Clonal analysis of human breast cancer by means of the polymerase chain reaction. *Cancer Res.*, **52**, 6594–6597.

NOWELL, P.C. (1986). Mechanisms of tumor progression. *Cancer Res.*, **46**, 2203–2207.

O'HARE, M.J. (1991). Breast cancer. In *Human Cancer in Primary Culture, A Handbook*, Masters, J.R.W. (ed.) pp. 271–286. Kluwer Academic Publishers: The Netherlands.

ÖRNDAL, C., MANDAHL, N., RYDHOLM, A., WILLÉN, H., CARLÉN, B., HEIM, S. & MITELMAN, F. (1993*a*). Cytogenetic heterogeneity and clonal evolution in a recurrent fibrosarcoma. *J. Exp. Clin. Cancer Res.*, **12**, 23–31.

ÖRNDAL, C., MANDAHL, N., WILLÉN, H., RYDHOLM, A. & MITELMAN, F. (1993*b*). Cytogenetic evolution in primary tumors, local recurrences, and pulmonary metastases of two soft tissue sarcomas. *Clin. Exp. Metastasis*, **11**, 401-408.

PANDIS, N., HEIM, S., BARDI, G., LIMON, J., MANDAHL, N. & MITELMAN, F. (1992*a*). Improved technique for short-term culture and cytogenetic analysis of human breast cancer. *Genes Chrom. Cancer*, **5**, 14-20.

PANDIS, N., HEIM, S., BARDI, G., IDVALL, I., MANDAHL, N. & MITELMAN, F. (1992*b*). Whole-arm t(1;16) and i(1q) as sole anomalies identify gain of 1q as a primary chromosomal abnormality in breast cancer. *Genes Chrom. Cancer*, **5**, 235-238.

PANDIS, N., HEIM, S., BARDI, G., IDVALL, I., MANDAHL, N. & MITELMAN, F. (1993*a*). Chromosome analysis of 20 breast carcinomas: cytogenetic multiclonality and karyotypic-pathologic correlations. *Genes Chrom. Cancer*, **6**, 51-57.

PANDIS, N., JIN, Y., LIMON, J., BARDI, G., IDVALL, I., MANDAHL, N., MITELMAN, F. & HEIM, S. (1993*b*). Interstitial deletion of the short arm of chromosome 3 as a primary chromosome abnormality in carcinomas of the breast. *Genes Chrom. Cancer*, **6**, 151-155.

PANDIS, N., JIN, Y., GORUNOVA, L., PETERSSON, C., BARDI, G., IDVALL, I., JOHANSSON, B., INGVAR, C., MANDAHL, N., MITELMAN, F. & HEIM, S. (1994*a*). Chromosome analysis of 97 primary breast carcinomas: identification of eight karyotypic subgroups (submitted).

PANDIS, N., BARDI, G. & HEIM, S. (1994*b*). Interrelationship between methodological choices and conceptual models in solid tumor cytogenetics. *Cancer Genet. Cytogenet.* (in press).

PETERSEN, O.W. & VAN DEURS, B. (1987). Preservation of defined phenotypic traits in short-term cultured human breast carcinoma derived epithelial cells. *Cancer Res.*, **47**, 856-866.

SAINT-RUF, C., GERBAULT-SEUREAU, M., VIEGAS-PÉQUIGNOT, E., ZAFRANI, B., CASSINGENA, R. & DUTRILLAUX, B. (1990). Proto-oncogene amplification and homogeneously staining regions in human breast carcinomas. *Genes Chrom. Cancer*, **2**, 18-26.

SCHMIDT-ULLRICH, R., LIN, P.S., MIKKELSEN, R.B. & MONROE, M.M. (1986). Proliferative rates of cloned malignant mammary epithelial cells as a measure of clonal heterogeneity in human breast carcinomas. *J. Natl Cancer Inst.*, **77**, 1001-1011.

SOBIN, L.H. (1981). *Histological Typing of Breast Tumours*, 2nd edn. WHO: Geneva.

THOMPSON, F., EMERSON, J., DALTON, W., YANG, J.M., McGEE, D., VILLAR, H., KNOX, S., MASSAY, K., WEINSTEIN, R., BHATTACHARYYA, A. & TRENT, J. (1993). Clonal chromosome abnormalities in human breast carcinomas. I. Twenty-eight cases with primary disease. *Genes Chrom. Cancer*, **7**, 185-193.

WAINSCOAT, J.S. & FEY, M.F. (1990). Assessment of clonality in human tumors: a review. *Cancer Res.*, **50**, 1355-1360.

WOLMAN, S.R. & HEPPNER, G.H. (1992). Genetic heterogeneity in breast cancer. *J. Natl Cancer Inst.*, **84**, 469-470.